# Epidemiology, Etiology, and Prevention of Late IOL-Capsular Bag Complex Dislocation: Review of the Literature

**DOI:** 10.1155/2015/805706

**Published:** 2015-12-21

**Authors:** Francisco J. Ascaso, Valentín Huerva, Andrzej Grzybowski

**Affiliations:** ^1^Department of Ophthalmology, University Clinic Hospital Lozano Blesa, Avenida San Juan Bosco 15, 50009 Zaragoza, Spain; ^2^Aragon Institute of Health Research (IIS Aragón), Avenida San Juan Bosco 13, 50009 Zaragoza, Spain; ^3^Department of Ophthalmology, University Hospital Arnau de Vilanova, Avenida Rovira Roure 80, 25198 Lleida, Spain; ^4^IRB Lleida, Avenida Rovira Roure 80, 25198 Lleida, Spain; ^5^Department of Ophthalmology, Poznan City Hospital, Ulica Szwajcarska 3, 61-285 Poznan, Poland; ^6^Department of Ophthalmology, University of Warmia and Mazury, 10-982 Olsztyn, Poland

## Abstract

Posterior chamber intraocular lens (PC-IOL) subluxation is uncommon but represents one of the most serious complications following phacoemulsification. Late spontaneous IOL-capsular bag complex dislocation is defined as occurring three months or later following cataract surgery. Unlike early IOL dislocation, late spontaneous IOL dislocation is due to a progressive zonular dehiscence and contraction of the capsular bag many years what seemed to be uneventful surgery. In recent years, late in-the-bag IOL subluxation or dislocation has been reported with increasing frequency, having a cumulative risk of IOL dislocation following cataract extraction of 0.1% after 10 years and 1.7% after 25 years. A predisposition to zonular insufficiency and capsular contraction is identified in 90% of reviewed cases. Multiple conditions likely play a role in contributing to this zonular weakness and capsular contraction. Pseudoexfoliation is the most common risk factor, accounting for more than 50% of cases. Other associated conditions predisposing to zonular dehiscence are aging, high myopia, uveitis, trauma, previous vitreoretinal surgery, retinitis pigmentosa, diabetes mellitus, atopic dermatitis, previous acute angle-closure glaucoma attack, and connective tissue disorders. The recognition of these predisposing factors suggests a modified approach in cases at risk. We review certain measures to prevent IOL-bag complex luxation that have been proposed.

## 1. Introduction

Cataract surgery with intraocular lens (IOL) implantation is a successful surgical procedure which has dramatically improved because of development in new techniques and devices, making it safer than it was two decades ago [1]. Sutureless clear corneal incision, continuous curvilinear capsulorhexis (CCC), phacoemulsification, and in-the-bag placement of a foldable IOL represent the gold standard for routine cataract surgery [2]. Although the surgical complication rate is low, anterior or posterior capsule opacification (PCO), capsule shrinkage or rupture, vitreous loss, and cystoid macular edema (CME) are some well-known complications of state-of-the-art cataract surgery [3].

Posterior chamber IOL subluxation or dislocation is uncommon but represents one of the most serious complications following cataract surgery. With respect to time, IOLs seem to be dislocated in a bimodal distribution. These dislocations are divided into early and late cases (Table 1) [4].

*Early IOL Dislocation*. This early complication often occurs due to improper IOL fixation within the secure capsular bag. Although zonular rupture may also be present preoperatively (e.g., in traumatic cataracts), dislocation is usually caused by tearing of the posterior capsule or rupture of the equatorial capsule and is often referred to as the sunset or sunrise syndrome [5, 6]. Furthermore, a zonular rupture is also believed to be a main cause of early IOL dislocation [1]. The zonules may be damaged during cataract surgery due to posterior pressure on the lens, either during a “can-opener style” capsulotomy or during nucleus phacoemulsification [7]. Finally, zonular rupture can occur even during IOL implantation [8]. Since the introduction of CCC with phacoemulsification the overall rate of IOL dislocation during the early postoperative period has decreased, as CCC gives 360° optic support and allows for better IOL fixation [9].

*Late Spontaneous IOL Dislocation*. It is defined as occurring three months or later following cataract surgery [1]. In contrast to early lens dislocation, bag dislocation generally occurs as a result of progressive zonular weakness many years after even uncomplicated cataract surgery, not from inadequate fixation of the IOL [5, 9]. Thus, the IOL is dislocated within an intact capsular bag many years after uneventful surgery in eyes that have had capsulorhexis [3, 4, 10–14]. IOL subluxation or dislocation within the capsular bag (“in-the-bag” IOL dislocation) differs from out-of-the-bag dislocation in the period of time between original cataract surgery and IOL dislocation, predisposing factors, and management [4]. Krėpštė et al. [1] retrospectively analyzed all the patients who were treated for late IOL dislocation requiring surgical management after routine cataract surgery was performed. They found that late IOL dislocation after phacoemulsification was mostly of the in-the-bag type, with late out-of-the-bag dislocation in only 12.1% of the cases.

## 2. Incidence and Distribution of Late IOL Dislocation

Even though the rate of posterior chamber IOL dislocation has been reported as 0.2% to 3% [15–17], late spontaneous dislocation is a small subset of this group [4]. The first case of late spontaneous in-the-bag IOL dislocation was described in 1993 by Davison [18] as a result of the capsule contraction syndrome. Since then, numerous isolated cases have been reported [3, 11, 19]. There are several reports of series of patients with spontaneous late IOL dislocation [3, 4, 9–11, 19, 20]. Although the exact incidence of this complication is not known, a survey of 2663 IOLs explanted between 1988 and 2001 demonstrated that “zonular dehiscence” was the reason for explantation in eight cases (0.3%) [21]. Nevertheless, this relatively small number represents only the tip of the iceberg, and 20% to 30% of cataract surgeons surveyed at the beginning of the 21st century reported this late-onset complication [22]. Furthermore, the incidence of late in-the-bag IOL dislocation has been rising since the popularization of the capsulorhexis and is most likely caused by factors such as zonular weakness and zonular stress that can occur during surgery or postoperatively in association with a moderate increase in postoperative anterior capsular fibrosis [3, 4, 9–14, 23].

In recent years, late in-the-bag IOL subluxation or dislocation has been reported with increasing frequency [3, 4, 9–12, 23, 24], leading to concerns of a pending large increase in IOL dislocations needing surgical intervention [3, 4, 11, 25, 26]. It is not clear if it is secondary to an increased rate of incidence of IOL dislocations or simply a larger community of at risk pseudophakic patients [27]. In 2009 and 2010, two population-based studies in Sweden estimated that the incidence of late IOL dislocation is low after phacoemulsification, but the authors were unable to significantly demonstrate an increased rate of incidence [14, 20]. More recently, a large retrospective, observational population-based cohort study identified a cumulative risk of IOL dislocation following cataract extraction of 0.1% after 10 years, 0.2% after 15 years, 0.7% after 20 years, and 1.7% after 25 years [27]. According to this study, the incidence of surgery specifically due to late dislocated IOL is 0.032–0.28% [20, 28]. However, the pseudophakic population in the Western world is growing rapidly as a result of the improvement in the quality and safety of phacoemulsification surgery, its expanded indications, the new phacorefractive procedures, and a longer lifespan. Therefore, the incidence of late IOL dislocation may still increase in the future [20, 29].

Late in-the-bag IOL subluxation or dislocation is a rare but serious complication due to progressive zonular dehiscence and contraction of the capsular bag many years after uneventful surgery. It is characterized by an IOL which is adequately fixed within the capsular bag. The entire lens-bag complex decenters with late dislocations. Zonular instability is the final common cause leading to within-the-bag dislocations [9]. Many of the spontaneous IOL dislocations occur several years after cataract surgery [29]. In fact, two-thirds of the reported cases occurred in the following two years, and the mean time interval between cataract extraction and repositioning surgery ranges from 6.9 to 8.5 years [4, 9, 11, 19, 20, 30, 31]. In pseudoexfoliation (PEX) cases the mean interval between cataract surgery and IOL dislocation is usually of 5.5–8.5 years [1, 3, 4, 7, 9, 10, 23, 29, 32], but some authors have treated cases presenting as late as 18 years after surgery [20]. Krėpštė et al. [1] found a significantly shorter interval in the eyes with a history of zonular laxity, cataract surgery complications, uveitis, and advanced or mature cataracts. Other authors observed that older age at cataract surgery and zonular dehiscence were significantly associated with a shorter time [20]. Because of the relatively long time frame for the presentation of this complication, an epidemic may occur in the future [1, 3].

According to Fernández-Buenaga et al. [29] the mean age of the patients at explantation surgery was 71.2 years (range 41–97). Likewise, patients in the high-myopia group were younger at time of explantation surgery than patients from the PEX group [4]. Most of the patients who underwent explantation due to late IOL dislocation were males (68.9%) [29]. This surprising result has also been found by other authors [9, 10, 14] and is difficult to explain because more women than men undergo cataract surgery [33] and have PEX [34]. Thus, it has been suggested that there may be a gender-related difference that results in weaker zonulae in men with PEX [29]. Nevertheless, it is not clear because not all papers which this review is based on found a gender difference.

Østern et al. [23] reported that 9.1% of the patients had bilateral late in-the-bag IOL dislocation, with a short period of time between both incidents. Bilaterality has also been observed by other authors [9, 12]. These findings suggest that, in patients with PEX, following IOL dislocation in one eye, it is important to pay particular attention to the unaffected eye [9, 12, 35].

## 3. Proposed Mechanisms in the Etiology of Late IOL Dislocation

Several mechanisms have been involved in postoperative capsule dislocation: preoperative trauma or zonular weakness, capsule contraction syndrome, and surgical or postoperative trauma to the zonules. The exact role and relative importance of each mechanism have not been widely agreed on and probably vary on a case-by-case basis [3].

### 3.1. Zonular Dehiscence

It often develops slowly over a long postoperative period because surgeons rarely report intraoperative phacodonesis [4]. Zonules are anchored by integrating in a mat-like fashion within the intrinsic fibers of the anterior and posterior capsules, approximately 2 mm anterior or posterior to the equator. A small subset of zonules inserts into the equator of the lens capsule, but they seem to bear a much smaller force load [36]. Zonules become more friable as patients age, especially in eyes with PEX, where there is a severe epithelial atrophy compared to non-PEX eyes of patients of the same age [37]. Zonular disruption anywhere along the course could cause zonulysis [4].

### 3.2. Contraction of the Capsular Bag

It may be present to some degree after cataract surgery. It happens as early as three months following phacoemulsification, but in the presence of solid zonule support does not lead to significant IOL displacement [10].

When capsular shrinkage is extreme, it is called “capsular contraction syndrome.” Such contraction results in additional stress on the potentially weakened zonules [9]. Although the advent of CCC made secure in-the-bag fixation popular, it can induce capsular fibrosis [3, 9]. Then, the sphincter effect of fibrosis around an intact CCC appears to be a factor in the development of significant capsule shrinkage [35]. In the presence of a very small CCC there is probably risk of IOL dislocation despite solid zonular support initially. Thus, CCC, particularly if its diameter is small, may be a significant risk factor for capsular contraction syndrome [3]. Some degree of capsular phimosis is frequent in most eyes following cataract surgery [38], but intense capsule shrinkage has only been described in cases with PEX [18, 39, 40], diabetes mellitus [10, 38], uveitis [18], pigmentary retinal degeneration [10], and myotonic dystrophy [41]. In patients with late IOL dislocation, the progressive weakening of already compromised zonules may make them vulnerable to continuous centripetal forces and cause their rupture [3].

### 3.3. Trauma

Although some reports consider that either preoperative or surgical trauma might be a cause of luxation [17, 42], no pseudophacodonesis is noted immediately after surgery in any reported case [3]. Furthermore, the contribution of neodymium:YAG (Nd:YAG) laser posterior capsulotomy to the late in-the-bag IOL dislocation syndrome is another obscure point. Although it has not been clarified yet, the impact of laser energy for treating posterior capsular opacification might be the triggering event for the subluxation [3, 4]. Likewise, the need for capsulotomy is an indicator of significant cell proliferation and of increased capsular bag weight. Because of this, it is reasonable to assume that, in eyes with fragile zonules, Nd:YAG capsulotomy could produce further loosening and should therefore, in these situations, either be carefully performed or be delayed until after secondary surgery [23]. Finally, major or minor postoperative trauma to the zonules (e.g., repeated eye rubbing) may contribute to bag dislocation [9, 43]. Indeed, Gimbel et al. [3] reported a known traumatic incident in 11.1% of patients.

## 4. Risk Factors

90% of reviewed cases show certain zonular weakness and capsular phimosis [44]. Although there are multiple predisposing factors, including aging [7], high myopia [25, 45], uveitis [4, 9, 18, 25, 46], trauma [4, 9, 43, 45, 47], previous vitreoretinal surgery [4, 7, 9, 46], retinitis pigmentosa [3, 9–11], diabetes mellitus [10], atopic dermatitis [43], previous acute angle-closure glaucoma attack [48], and connective tissue disorders, such as Marfan's syndrome, homocystinuria, hyperlysinemia, Ehler-Danlos syndrome, scleroderma, and Weill-Marchesani syndrome [39], PXF is the most common risk factor, accounting for more than 50% of cases [1, 3, 4, 7, 9–12, 20, 27, 29, 32, 35, 49–51]. All these factors seem to increase the risk of zonular weakness and capsular contraction [9–11].

We have reviewed the main risk factors predisposing to zonular instability and capsular contraction: PEX and high myopia.

### 4.1. Pseudoexfoliation (PEX)

PEX syndrome is a pathological condition consisting of a meshwork of abnormal fibrillar material, deposited on the lens surface and into all structures in the anterior chamber [50]. These accumulations may both mechanically and enzymatically damage the zonules, weaken their points of anchorage to the ciliary body and lens [52, 53], and facilitate the anterior capsule contraction syndrome that, if left untreated, usually leads to zonular failure [18, 40]. PEX has always been the most recognized predisposing factor for late dislocation [4, 11, 35].

PEX is also thought to have a genetic basis associated with lysyl oxidase-like 1 (LOXL1) allelic variants [54]. LOXL1 is a member of a gene family that plays an important role in elastin metabolism [55].

Liu et al. have recently provided a complete histopathologic analysis of explanted capsular bags that are spontaneously dislocated in the late postoperative period [55]. These authors demonstrated that PXF material is present in a larger proportion of late in-the-bag IOL subluxations and dislocations than the number currently detected clinically, as a result of significant clinical underdiagnosis. Indeed, PEX can be a difficult clinical diagnosis, with many subclinical cases going unnoticed until well advanced [56–58].

The incidence of PEX varies widely according to geographical location and ethnicity [59–61]; therefore, the incidence of in-the-bag dislocation is expected to vary accordingly [19]. Certain studies have evaluated the rate of PXF in specific populations, finding a higher incidence (25% to 30%) in some ethnic groups, such as northern Scandinavians [62, 63], Saudi Arabians [64], and Navajo Indians [13]. Liu et al. [55] demonstrated a nearly 2 : 1 female predilection.

Østern et al. [23] demonstrated that long after phacoemulsification surgery (6-7 years), IOLs were positioned significantly lower in PEX patients than in controls, suggesting zonular weakness in at least some of them.

### 4.2. High Myopia

High myopia is a well-known risk factor for late IOL dislocation [45, 65, 66]. Nevertheless, only one article presented this condition as the main risk factor for late spontaneous in-the-bag IOL dislocation, finding it in 19.7% of the cases [29]. High-myopic eyes show some typical alterations due to thinning and degeneration of several eye layers as lacquer cracks, chorioretinal atrophy, or posterior staphyloma [67]. It has been hypothesized that, as well as the previously mentioned alterations, these eyes may be also more prone to zonular failure due to excessive elongation of the zonular fibers, which have to support greater stress than in emmetropic eyes [68, 69].

## 5. Prevention

The recognition of risk factors for this complication suggests a modified approach in cases at risk [3]. Certain measures to prevent IOL-bag complex luxation have been proposed [70]. Thus, CCC diameter should be smaller than the optic [2], but a particularly small opening should be avoided [22], because it increases capsule fibrosis and shrinkage [38]. If capsulorhexis fibrosis and contracture are detected, relaxing cuts with Nd:YAG laser should be performed.

During phacoemulsification, caution should be given to keeping the integrity of zonules. Chopping techniques are less traumatic to the zonules. Aspiration of cortex directed in a tangential fashion rather than perpendicular to the zonules may decrease the incidence of zonular rupture. Meticulous cortex cleaning is advocated in all cases. This may be technically difficult in eyes with PEX due to the small pupil, poor resistance by the zonules, and possible lens subluxation [70].

Particular attention should be paid to implanting a posterior chamber IOL in the capsular bag in eyes with weakened zonules when progressive zonular disruption is anticipated. We plan haptics in the sulcus and IOL capture through an anterior capsulorhexis preoperatively in eyes with risk factors such as PEX, retinitis pigmentosa, uveitis, and long axial length without a capsule tear and vitreous loss.

IOL material and design may affect capsular contraction and IOL decentration [3]. Single-piece poly(methyl methacrylate) (PMMA) IOLs may counteract capsule contraction better than 3-piece PMMA IOLs [9, 25]. It has also been suggested that a 3-piece hydrophobic acrylic IOL may reduce CCC shrinkage through a combination of decreased anterior capsule fibrosis and greater haptic rigidity [22, 35]. Several authors consider that 1-piece acrylic IOLs may produce greater capsular contraction or offer less haptic resistance to contraction than 3-piece acrylic lenses. It is well known that silicone induces much more capsular fibrosis and risk of IOL dislocation. There is no doubt that plate-haptic silicone IOLs induce the most capsulorhexis contracture, suggesting they may be contraindicated in high-risk cases [35].

Although there is no proof of the use of CTRs to prevent IOL dislocation, theoretically, the routine use of capsular tension rings (CTRs) seems to provide a reasonable preventive measure, since there is evidence on its role in preventing zonular loss during surgery [8, 11, 70]. They would be indicated when there is zonular instability following surgical or postoperative trauma or in cases of inherently weak zonules, as in PEX syndrome [71–73]. Moreover, CTRs may prevent intraoperative zonular dehiscence [74] and decrease [75] but not avoid [76–78] postoperative capsule shrinkage. CTRs may also prevent capsular folds and, in that way, reduce the rate of posterior capsular opacification [2, 71]. In the absence of significant zonular rupture, routine CTR implantation in cases at risk may diminish the incidence of postoperative IOL decentration due to the resistance to capsular contraction [79]. Furthermore, a CTR may facilitate secondary suturing of a dislocated IOL.

Postoperative pseudophacodonesis should be monitored closely because this may evolve to complete luxation [49]. Finally, Nd:YAG laser posterior capsulotomy should be carefully performed in PEX eyes before any visible capsular contraction.

## Figures and Tables

**Table 1:** Types of spontaneous IOL dislocations.

	Early cases	Late cases
Time following cataract surgery	<3 months	≥3 months (even years after uncomplicated cataract surgery)

Pathogenesis	Inadequate IOL fixation within the secure capsular bag	Progressive zonular insufficiency and capsular bag contraction

Predisposing factors	Tearing of the posterior capsule and rupture of the equatorial zonule	Aging, high myopia, uveitis, trauma, retinitis pigmentosa, diabetes mellitus, atopic dermatitis, connective tissue disorders, and previous vitreoretinal surgery or acute angle-closure glaucoma attack
